# Development of a Culturally Adapted Intervention on Feeding Options for Chinese American Dementia Caregivers

**DOI:** 10.1093/geroni/igaf122.1124

**Published:** 2025-12-31

**Authors:** Yaolin Pei, Jing Wang, Xiang Qi, Dena Schulman-Green, Laura Hanson, Bei Wu

**Affiliations:** The University of Texas at Austin, Austin, Texas, United States; University of New Hampshire, Durham, New Hampshire, United States; New York University, New York, New York, United States; New York University, New York, New York, United States; The University of North Carolina at Chapel Hill School of Medicine, Chapel Hill, North Carolina, United States; NYU Shanghai, Shanghai, China (People’s Republic)

## Abstract

Considering Chinese American dementia caregivers’ strong preference for tube feeding and use of a family decision-making model regarding end-of-life care, it is crucial to provide culturally adapted decision support. This qualitative descriptive study aimed to inform the cultural adaptation of an existing proven decision aid intervention for Chinese American dementia families. We conducted semi-structured, in-depth interviews with 21 past and current Chinese American dementia caregivers and 13 health care providers (i.e., physicians, social workers, nurses, and speech-language pathologists). Individual interviews were audio-recorded, transcribed, and thematically analyzed. Guided by the Ecological Validity Model, which specifies eight domains of cultural adaptation (language, persons, metaphors, content, concepts, goals, methods, and context), we culturally adapted the decision aid intervention. For example, qualitative findings revealed that family harmony is central to decision-making on feeding options. To address this, the intervention’s goal was refined to promote family consensus while improving informed decision-making. In the “content” domain, we add the strategies for family communication and encourage participants to discuss the decision-making on feeding options with other family members. Additionally, misconceptions equating careful handing feeding with starvation were identified. Thus, the decision aid clarifies that individuals with advanced dementia have reduced nutritional needs and distinguished this from starvation. The cultural adaptation of the decision aid intervention serves as a model to align with cultural values, address common misconceptions, and facilitate informed, family-centered decision-making in dementia care. In the next step, we will use cognitive interviews and field test to refine this culturally adapted decision aid intervention.

